# Self-Advantage in the Online World

**DOI:** 10.1371/journal.pone.0140654

**Published:** 2015-10-13

**Authors:** Hongsheng Yang, Fang Wang, Nianjun Gu, Ying Zhang

**Affiliations:** 1 School of Psychology, Southwest University, Chongqing, China; 2 Research Center of Psychology and Social Development; Southwest University, Chongqing, China; Centre de Neuroscience Cognitive, FRANCE

## Abstract

In the current research, screen name was employed to explore the possible cognitive advantage for self-related online material. The results showed that one’s own screen name and real name were detected faster than famous names in both visual search and discrimination tasks. In comparison, there was no difference in visual search speed for the two kinds of self-related names. These findings extend self-advantage from the physical world to the virtual online environment and confirm its robustness. In addition, the present findings also suggest that familiarity might not be the determining factor for self-advantage.

## Introduction

Cognitive advantage for self-related information has been established in a large number of studies using face and name stimuli. For example, one’s own face demonstrates consistently faster response time in a visual search task than faces of others. In addition, it is also difficult to ignore when used as distracter item and show strong interfering power on target stimuli [1,2]. Research using name stimuli provides another line of evidence for this advantage. Among them is the well-known cocktail party effect [3]. That is, due to its personal significance, one’s name is intrinsically meaningful to its owner and can easily capture one’s attention, even in unattended conditions [4–7].

According to Keenan et al., (1999), there may be a general self-advantage [8]. Research has shown that one’s own face and name can elicit similar brain potential and response time when compared directly with each other [9]. However, the stimuli employed in previous studies are mostly faces and names. As stable signs of personal identity, these two kinds of stimuli are highly familiar and cannot be easily changed in most cases as they are either determined biologically or chosen by family members. On the other hand, the fact has been largely neglected that there are many materials which are personally-relevant yet also alterable, thus not as familiar to the owners as face and name. That is, they can choose or modify these materials by themselves. For example, one can choose, change, or even delete his/her screen name and avatar on the Internet. Whether the same cognitive advantage for one’s own face and name can exist for these fluid self-related materials is an interesting issue as it can be helpful to extend the research scope of self-advantage and examine its robustness. In addition, another issue related to cognitive advantage for self-related information that needs further investigation is the extent to which familiarity might influence this advantage. Given the high familiarity inherent in self-related information, it seems quite reasonable to attribute this advantage to familiarity in some way. Surprisingly, little research has been done to address this issue in the field of one’s own name recognition. The few studies that have employed familiar names as control stimuli only focused on their neural mechanism and still yielded inconsistent results [10–12].

Based on the literature reviewed, the current research was designed to examine the cognitive advantage for self-related information using a kind of novel stimuli—screen name (also known as online username or nickname). In addition to extending research on self-advantage, since one’s own screen name is theoretically less familiar than one’s own real name, the current research also explored the effect of familiarity on self-advantage by making comparisons between these two self-relevant names with different familiarity.

Despite their difference in formation rules and usage practices, screen names share strong functional similarity with real names in their respective context. Just like real names in the physical world, screen names are also used as personal signs in the online world where they provide an important way for people to represent themselves. Although they can be very fluid and are easily and quickly changed to meet users’ current needs or desires, Bechar-Israeli (1995) found that screen names are often chosen just as carefully as real names and if forced to change from a favored one, the new screen names may still maintain strong ties to their earlier alternatives [13]. Therefore, screen names often bear strong significance and act as highly personal markers for individuals on the Internet [14]. In this sense, they are just like real names. Given the functional similarities between a screen name and a real name as well as their common self-relevance, it was expected that a similar cognitive advantage may exist for one’s own screen names in the online world. Two experiments were conducted to explore this possibility in the current research.

## Experiment 1

In this experiment, the task of participants was to search for their real name, screen name or one famous name in separate blocks of trials.

### Method

#### Ethical Statement

This study was approved by The Ethics Committee of Southwest University. All participants provided written consent and were paid for their participation.

#### Participants

Thirty college students (20 females and 10 males; mean age = 20.9 ± 1.43 years) with normal or corrected-to-normal vision participated in this experiment. All participants had a favorite screen name composed of two or three Chinese characters (mean usage time was 3.97 years; participants spent an average of 11.2 hours per week using it in online activities).

#### Stimuli

The stimuli included participants’ own real name, their screen name, and one famous name plus 100 other names and 100 other screen names which served as distracter stimuli. All real names and screen names were matched for length and number of character strokes through pre-experimental screening. Famous names were drawn from pop stars, writers, politicians, and other well-known persons. Two weeks before the experiment, participants were provided one list of these names and asked to choose one which they were familiar with. Stimuli to be used as distracters were also presented for them to exclude those they might know.

In each trial, two, six, or twelve names were presented in black against a light-grey background. In 12-name displays, stimuli were evenly dispersed around a central cross (the fixation point) and formed a virtual circle of 20 cm. in diameter (visual angle about 15.2 degrees, based on viewing distance of 75 cm.). For displays with two or six screen names, stimuli positions were selected randomly from the total set of 12 positions. There was a constraint for all the three types of displays that the frequency target name that appeared in each of the 12 positions was kept equal. For each display, half of the stimuli were real names and the other half were screen names. Distracter items were chosen randomly from the total list of 200 distracter real and screen names.

#### Procedure

The experiment consisted of three blocks, each including 288 trials and requiring participants to search for one specific target (own real name, own screen name, or one famous name). The order of different blocks was counterbalanced across participants. A 3×3 within-subject design was employed in which participants were required to search for one of the three targets in displays of three set size (2, 6, or 12 names). Within each block, half of the trials contained the target, the other half did not, and there were forty-eight trials in each of the six combinations of target presence/absence by display set size.

**Fig 1:**
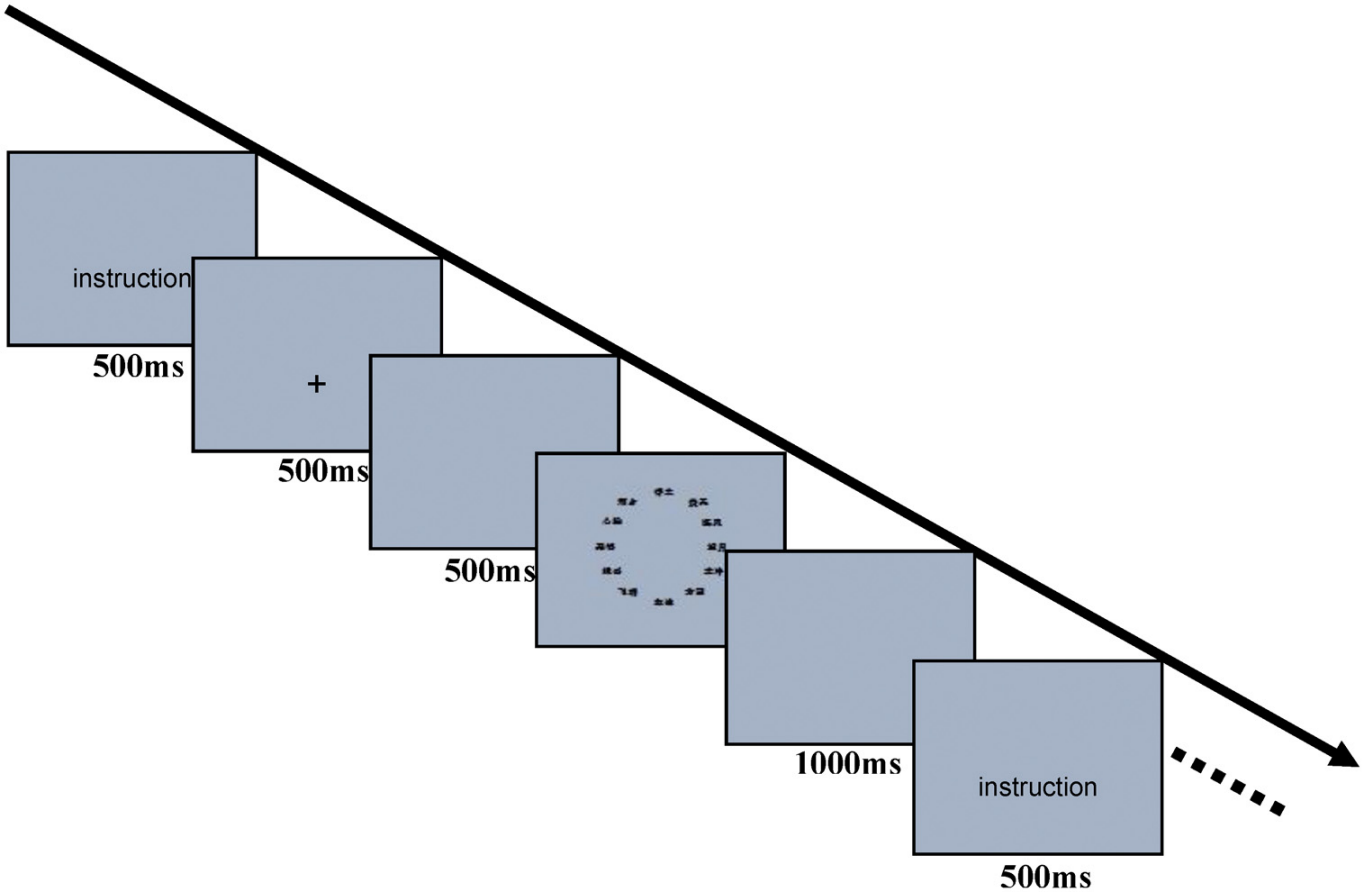
The flow chart for procedure of Experiment 1.

The procedure was shown in Fig 1. Each trial began with an instruction for preparation and a 500 ms blank screen. Next, a cross was presented in the center of the screen for 500 ms, followed by 500 ms of blank screen, After that, the stimuli names were given and remained present until participants gave their response. A 1 s pause was interposed between adjacent trials. Participants were required to respond as quickly and accurately as possible. All participants used their right index and middle finger to make yes/no responses.

### Results and analysis

Within each level combination of target type and display set size, response time exceeding three standard deviations (SD) above or below the mean were removed. False alarm rates for the three targets (own real name: 0.7%; own screen name: 0.7%; famous name: 0.8%) were compared and demonstrated no significant difference, F(2, 58) = 0.035, p > .05.

Table 1 shows the mean response time for one’s own real name, screen name, and famous name in three different display set sizes. These data were submitted to a repeated 3×3 ANOVA. Main effects for target type and display set size were both significant, target type: F (2, 58) = 11.58, p < .001, η_*p*_^2^ = .29; display set size: F(2, 58) = 346.79, p < .001, η_*p*_^2^ = .92. There was also a significant target type × display set size interaction, F(4, 116) = 6.54, p < .001, η_*p*_^2^ = .18. Analysis of simple main effects showed that regardless of display size, there was no RT difference between one’s own screen name and real name, with a 95% confidence interval of -36.116 to 19.427, -90.607 to 39.943, -246.983 to 42.846, p = .54, .43, .16 respectively for trials of 2, 6 and 12 stimuli. In comparison, one’s own real name was detected consistently faster than famous name, all ps < .001. Like one’s own real name, screen name also demonstrated faster search speed than famous name in trials with 2, 6, or 12 items, p< .01, .001, and .05 respectively. On the other hand, response time for each kind of target was monotonically higher as the display set size increased, all ps < .001.

**Table 1:** Response time (ms) for one’s own name, screen name, and famous name under different display set size.

	Display set size		
	2	6	12
Own name	721(83)	951(158)	1233(275)
Own screen name	729(88)	976(168)	1335(388)
Famous name	775(76)	1094(143)	1505(239)

Analysis of accuracy data also demonstrated a significant difference, F(2, 58) = 6.64, p < .01, η_*p*_^2^ = .19. Hit rate for own real name (97.4%) was higher than that both for own screen name (96.2%) and famous name (95.0%), p < .05 and p < .01 respectively. There was no difference between own screen name and famous name, p >.05.

## Experiment 2

In Experiment 1, one’s own name, screen name and famous name were arranged into different blocks so that participants needed to search for them separately. The results showed that one’s own screen name has a similar cognitive advantage with one’s own real name even under the distraction of other real names. Experiment 2 employed a visual discrimination task in which all the three target names were presented centrally within a single block and participants were asked to discriminate them from other distracter names. The aim was to examine if one’s own screen name can still demonstrate faster response time when the target names were presented within the focus of attention.

### Method

#### Participants

Thirty right-handed college students (21 females, 9 males; mean age: 20.7 ± 1.6) participated in this experiment. All participants had a favorite screen name composed of two or three Chinese characters (mean usage time was 4.8 years; they spent an average of 20.3 hours per week using it in online activities). None of them had participated in the first experiment or had any previous experience of such experiments.

#### Stimuli

The stimuli were the same as those used in Experiment 1.

#### Procedure

This experiment consisted of 360 trials. In each trial, one name or screen name was presented in the center of screen and participants were asked to give a response based on the identity of this name. If it was their own name, screen name, or the famous name, they were asked to respond positively by pressing one designated key. If not, they were asked to press another key. During the experiment, each of the three names was repeated 60 times. In addition, 90 screen names and 90 real names served as distracter items. The order of different names was randomized.

### Results and analysis

**Fig 2:**
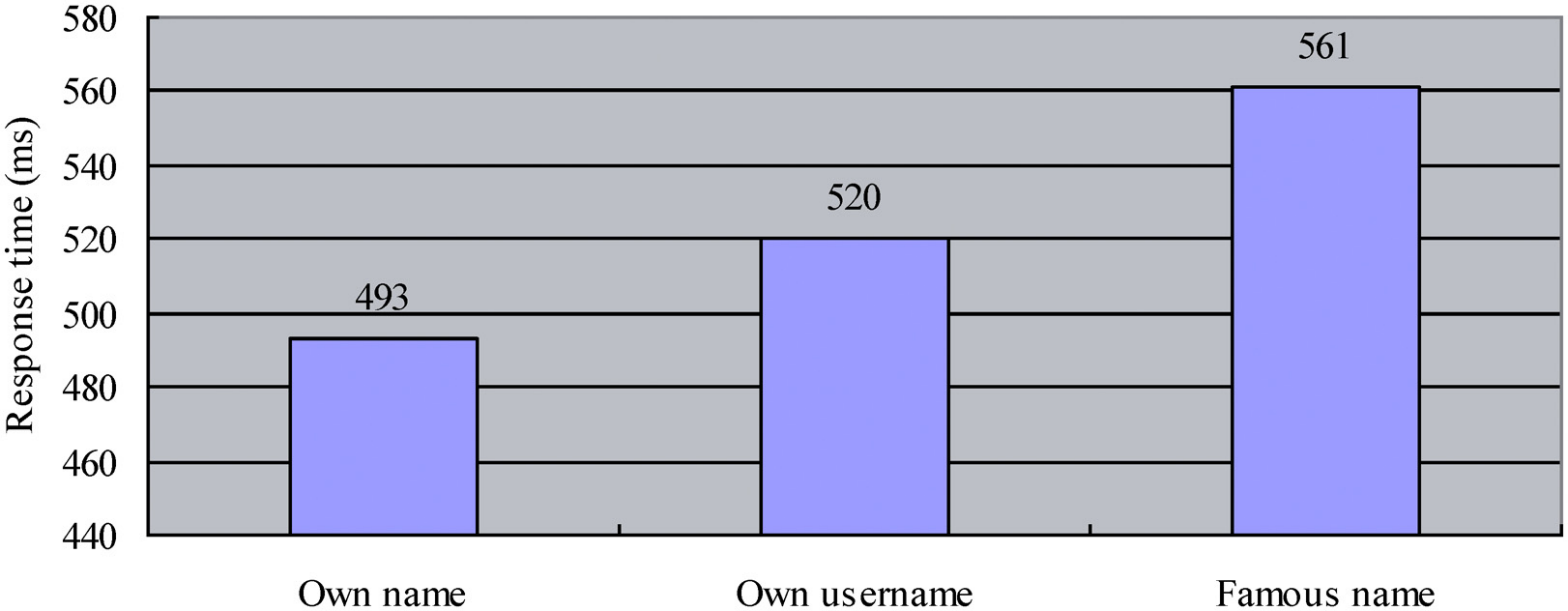
Response time for one’s own name, screen name, and famous name.

Response time exceeding three standard deviations (SD) above or below the mean for each kind of name was removed. The results were shown in Fig 2. A repeated measures ANOVA found a significant difference in discrimination speeds for the three names, F(2, 58) = 117.65, p < .001, η_*p*_^2^ = .80. Post hoc analysis showed that RT for own real name (M = 493 ms, SD = 77) was shorter than that for either own screen name (M = 520 ms, SD = 73) or the famous name (M = 561 ms, SD = 72), ps < .001. More importantly, replicating the results from Experiment 1, own screen name demonstrated similar advantage over the famous name as it was detected faster than the latter, p < .001. In addition to the differences in response time, accuracy data for the three names were also different, F(2, 58) = 12.79, p < .001, η_*p*_^2^ = .31. Hit rates for both own real name (M = 99.4%) and screen name (M = 98.4%) were higher than that for the famous name (M = 96.3%), p < .001 and .01 respectively, while there was no difference between the two kinds of self-related names, p > .05.

## General Discussion

Previous electrophysiological studies have confirmed that “fluid” self-relevant materials/information such as one’s own objects, name of hometown or high school, and et al. can evoke differential ERP from others-related materials [15,16]. In a similar vein, the current research showed that one’s own screen name can elicit faster response times than a famous name in both visual search and discrimination tasks. More strikingly, the self-related online material demonstrated similar visual search speed with one’s own name even when there were real names serving as distracter items. These results confirmed a cognitive advantage for one’s own screen name and provided support for the notion of a general self-advantage [8]. Based on the existence of such an online cocktail party effect, self-advantage can be extended from the real world to the virtual online environment.

It is beneficial to compare the similarities and differences between screen names and real names to understand the cognitive advantage for one’s own screen names. Unlike real names which are usually chosen and fixed from parents or other respected persons, screen names are mostly self-generated or selected based on personal interests and can be changed easily. Moreover, screen names have a relatively shorter history of usage compared with real names. For example, the mean usage time of screen names was only 3.97 and 4.8 years for participants in the current two experiments. In comparison with these large differences, the only apparent commonality shared by the two kinds of names is their function as personal markers in respective usage environment. As a result, the common self-relevance might be the underpinning factor for their similar visual search performance and common advantage over a famous name.

As has been revealed through content analysis, screen names are directly related to the self in many cases [13,14]. According to Stommel, formation of screen names can be viewed as a process of self-construction. They are not just a symbol which simply marks the existence of one Internet user, but moves beyond this to constitute an important form of self representation in the virtual world. Due to this kind of self-relevance, one’s own screen name holds a strong personal salience.

An ERP study showed that when participants were asked to respond positively to an assumed “own” name and reject as false all other names, including the true name, the evoked brain potential to the assumed name was more different from that elicited by other false names than one’s true name [17]. Like the assumed name, screen names employed in the current research are just common words and have no special meaning to its owner before they are chosen as an online ID. Nonetheless, when they were connected with the self, even temporarily, both demonstrated their capacity to facilitate cognitive processing. Taken together, these findings demonstrated the robustness of self-advantage.

In addition to confirming the cognitive advantage for one’s own screen name, the present findings also suggested that familiarity may not be the determining factor for self-advantage as both one’s own real name and screen name showed faster response time in visual search and discrimination task when compared with famous names. However, it should be noted that own real name was detected faster than own screen name in Experiment 2, which suggested that familiarity still plays some role in self-advantage and the differentiation between self-relevant and close other-relevant material may depend on the specific measures/tasks [16].
